# New *M* ^+^, *M* ^3+^-arsenates – the framework structures of Ag*M* ^3+^(HAsO_4_)_2_ (*M* ^3+^ = Al, Ga) and *M* ^+^GaAs_2_O_7_ (*M* ^+^ = Na, Ag)

**DOI:** 10.1107/S2056989017005631

**Published:** 2017-04-28

**Authors:** Karolina Schwendtner, Uwe Kolitsch

**Affiliations:** a Institute for Chemical Technology and Analytics, Division of Structural Chemistry, TU Wien, Getreidemarkt 9/164-SC, 1060 Wien, Austria; b Naturhistorisches Museum Wien, Burgring 7, 1010 Wien, Austria; c Institute for Mineralogy and Crystallography, University of Vienna, Althanstrasse 14, 1090 Wien, Austria

**Keywords:** crystal structure, AgAl(HAsO_4_)_2_, AgGa(HAsO_4_)_2_, AgGaAs_2_O_7_, NaGaAs_2_O_7_

## Abstract

The crystal structures of hydro­thermally synthesized silver(I) aluminium bis­[hydrogen arsenate(V)], AgAl(HAsO_4_)_2_, silver(I) gallium bis­[hydrogen arsenate(V)], AgGa(HAsO_4_)_2_, silver gallium diarsenate(V), AgGaAs_2_O_7_, and sodium gallium diarsenate(V), NaGaAs_2_O_7_, were determined from single-crystal X-ray diffraction data collected at room temperature. The first two compounds are representatives of the MCV-3 structure type known for KSc(HAsO_4_)_2_, which is characterized by a three-dimensional anionic framework of corner-sharing alternating *M* ^3+^O_6_ octa­hedra (*M* = Al, Ga) and singly protonated AsO_4_ tetra­hedra. Inter­secting channels parallel to [101] and [110] host the Ag^+^ cations, which are positionally disordered in the Ga compound, but not in the Al compound. The hydrogen bonds are relatively strong, with O⋯O donor–acceptor distances of 2.6262 (17) and 2.6240 (19) Å for the Al and Ga compounds, respectively. The two diarsenate compounds are representatives of the NaAlAs_2_O_7_ structure type, characterized by an anionic framework topology built of *M* ^3+^O_6_ octa­hedra (*M* = Al, Ga) sharing corners with diarsenate groups, and *M* ^+^ cations (*M* = Ag) hosted in the voids of the framework. Both structures are characterized by a staggered conformation of the As_2_O_7_ groups.

## Abstract 2

The crystal structures of hydro­thermally synthesized AgAl(HAsO_4_)_2_, AgGa(HAsO_4_)_2_, AgGaAs_2_O_7_ and NaGaAs_2_O_7_ are reported. The first two compounds are representatives of the MCV-3 structure type, which is characterized by a three-dimensional anionic framework of corner-sharing alternating *M* ^3+^O_6_ octa­hedra and singly protonated AsO_4_ tetra­hedra. Inter­secting channels parallel to [101] and [010] host the Ag^+^ cations. The two diarsenate compounds are representatives of the NaAlAs_2_O_7_ structure type, characterized by an anionic framework topology built of *M* ^3+^O_6_ octa­hedra sharing corners with diarsenate groups, and *M* ^1+^ cations hosted in the voids of the framework.

## Chemical context   

Metal arsenates often form tetra­hedral–octa­hedral framework structures with potentially inter­esting properties, such as ion conductivity, ion exchange and catalytic properties (Masquelier *et al.*, 1990 ▸, 1994*a* ▸,*b* ▸, 1995 ▸, 1996 ▸; Medvedev *et al.*, 2003 ▸; Ouerfelli *et al.*, 2007*a* ▸, 2008 ▸; Pintard-Scrépel *et al.*, 1983 ▸; Rousse *et al.*, 2013 ▸). A detailed study of the system *M* ^+^–*M* ^3+^–As–O–(H) was therefore conducted, and a wide variety of new compounds and structure types were found and have been published (Schwendtner, 2006 ▸; Schwendtner & Kolitsch, 2004*a* ▸,*b* ▸, 2005 ▸, 2007*a* ▸,*b* ▸,*c* ▸,*d* ▸). Thus far, three different structure types for *M* ^1+^ *M* ^3+^(HAsO_4_)_2_ compounds have been reported. Two of them are very common and were first described for isotypic phosphate compounds. The (H_3_O)Fe(HPO_4_)_2_ type (Vencato *et al.*, 1989 ▸) is also adopted by β-CsSc(HAsO_4_)_2_ (Schwendtner & Kolitsch, 2004*b* ▸), while the (NH_4_)Fe(HPO_4_)_2_ type (Yakubovich, 1993 ▸) is adopted by α-CsSc(HAsO_4_)_2_ (Schwendtner & Kolitsch, 2004 ▸) and (NH_4_)Fe(HAsO_4_)_2_ (Ouerfelli *et al.*, 2014 ▸). The KSc(HAsO_4_)_2_ type (Schwendtner & Kolitsch, 2004*a* ▸) has so far been the only known example; the two new protonated arsenates presented here are two further representatives of this structure type.

*M* ^+^ *M* ^3+^As_2_O_7_ compounds crystallize in six known structure types, some of them isotypic to phosphates or silicates. The CaZrSi_2_O_7_ type (mineral gittinsite; Roelofsen-Ahl & Peterson, 1989 ▸) is also adopted by LiFeAs_2_O_7_ (Wang *et al.*, 1994 ▸), Li*M* ^3+^As_2_O_7_ (*M* ^3+^ = Al, Ga, Sc) and NaScAs_2_O_7_ (Schwendtner & Kolitsch, 2007*d* ▸). The NaInAs_2_O_7_ type (Belam *et al.*, 1997 ▸) has no other known members. The TlInAs_2_O_7_ type (*M* ^+^ = Tl, Rb, NH_4_) (Schwendtner, 2006 ▸) is also adopted by KFeAs_2_O_7_ (Ouerfelli *et al.*, 2007*b* ▸). The RbAlAs_2_O_7_ type (Boughzala *et al.*, 1993 ▸) is also known for *M* ^1+^ = Tl, Cs (Boughzala & Jouini, 1992 ▸), *M* ^1+^ = K (Boughzala & Jouini, 1995 ▸), and is further represented by KGaAs_2_O_7_ (Lin & Lii, 1996 ▸), KCrAs_2_O_7_ (Siegfried *et al.*, 2004 ▸) and the mixed (Al/Fe) compound TlAl_0.78_Fe_0.22_As_2_O_7_ (Ouerfelli *et al.*, 2007*a* ▸). The KAlP_2_O_7_ type is extremely common for phosphates and also has five examples that are arsenates, *M* ^+^ScAs_2_O_7_ with *M* ^+^ = NH_4_ (Kolitsch, 2004 ▸), Rb (Schwendtner & Kolitsch, 2004*a* ▸), and Tl (Baran *et al.*, 2006 ▸), as well as CsCrAs_2_O_7_ (Bouhassine & Boughzala, 2015 ▸), and the mixed (Al/Cr) compound K(Al_0.75_Cr_0.25_)As_2_O_7_ (Bouhassine & Boughzala, 2017 ▸). The NaAlAs_2_O_7_ type (Driss & Jouini, 1994 ▸) is also known for AgFeAs_2_O_7_ and NaFeAs_2_O_7_ (Ouerfelli *et al.*, 2004 ▸), and the *M*1^2+^ *M*2^2+^ representative CaCuAs_2_O_7_ (Chen & Wang, 1996 ▸). The two diarsenates presented here also adopt the NaAlAs_2_O_7_ structure type.

## Structural commentary   

**Figure 1:**
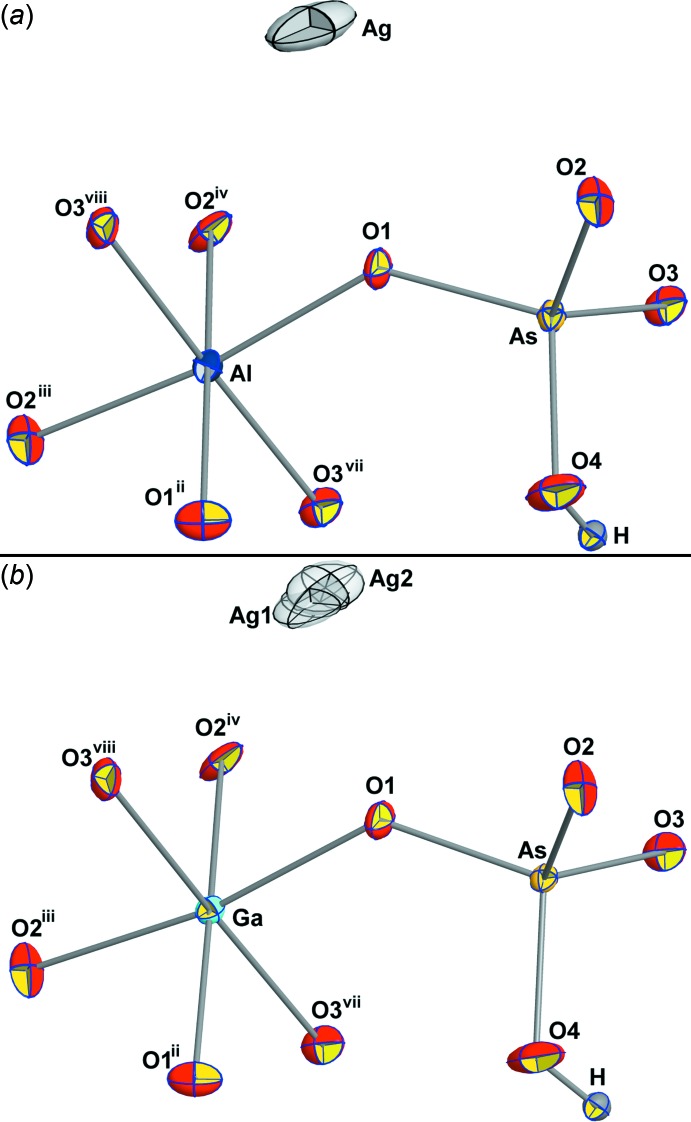
The principal building units of (*a*) AgAl(HAsO_4_)_2_ and (*b*) AgGa(HAsO_4_)_2_, shown as displacement ellipsoids at the 70% probability level. The H atom is shown as a sphere with arbitrary radius. Part (*b*) shows the split position for Ag in AgGa(HAsO_4_)_2_. [Symmetry codes: (ii) −*x*, *y*, −*z* + 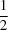; (iii) *x* + 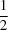, *y* + 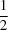, *z*; (iv) −*x* + 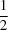, *y* + 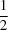, −*z* + 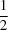; (vii) −*x* + 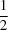, −*y* + 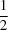, −*z*; (viii) *x* + 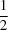, −*y* + 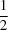, *z* − 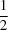.]

**Figure 2:**
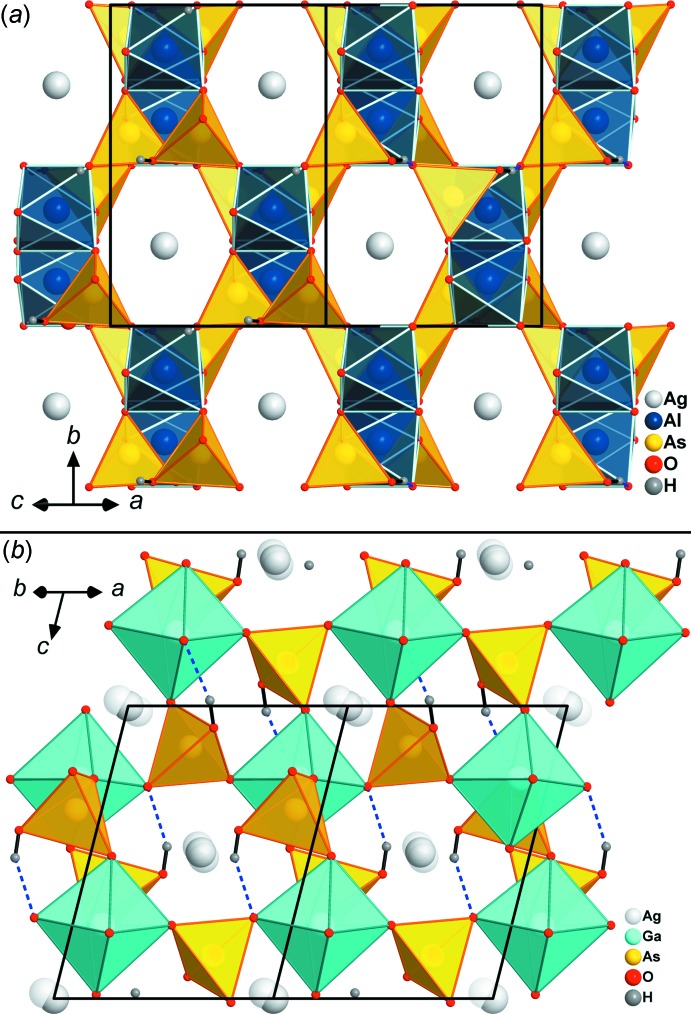
View of the framework structure of (*a*) AgAl(HAsO_4_)_2_ along [101] and (*b*) isotypic AgGa(HAsO_4_)_2_ along [110]. The *M* ^3+^O_6_ octa­hedra (*M* = Al, Ga) are corner-linked to HAsO_4_ tetra­hedra. Both views show small micropores in which the Ag^+^ cations are situated; the split Ag position (see text) and hydrogen bonds (dashed lines) are indicated in (*b*).

AgAl(HAsO_4_)_2_ and AgGa(HAsO_4_)_2_ are isotypic and crystallize in the monoclinic (*C*2/*c*) microporous framework structure of KSc(HAsO_4_)_2_ (Schwendtner & Kolitsch, 2004*a* ▸). The asymmetric unit contains one Ag, one Ga/Al, one As, four O and one H sites (Fig. 1 ▸). The Al/Ga atoms lie on a special position (twofold rotation axis) and the Ag^+^ cation is situated on an inversion centre. In the case of AgGa(HAsO_4_)_2_, the Ag^+^ cation occupies a split position (Fig. 1 ▸ *b*), where Ag1 sits on the inversion centre, with a freely refined occupancy of 0.75 (2). The second site (Ag2) is only 0.31 (3) Å away from Ag1 and has a freely refined occupancy of 0.123 (10). In total, this leads to a composition of one Ag atom per formula unit. The slightly distorted *M* ^3+^O_6_ octa­hedra share corners with six hydrogen arsenate tetra­hedra to form a three-dimensional, anionic framework structure with narrow irregular channels parallel to [101] and [110] (Fig. 2 ▸), which host the Ag^+^ cations. The latter show a rather irregular [6 + 2]-coordination in both compounds. However, it is well known that Ag atoms tolerate a broad spectrum of coordination spheres (Müller-Buschbaum, 2004 ▸).

The protonated apex of the AsO_4_ tetra­hedron is involved in a relatively strong hydrogen bond with O4⋯O2(*x*, −*y* + 1, *z* + 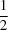) = 2.6212 (17) and 2.6240 (19) Å for the Al (Table 2 ▸) and Ga compounds (Table 4 ▸), respectively, which runs roughly perpendicular to the (101) plane (Fig. 2 ▸). The As—OH bond lengths (Tables 1 ▸ and 3 ▸) are considerably elongated in comparison to the three remaining As—O bonds (Ferraris & Ivaldi, 1984 ▸), but at 1.7161 (10) Å for the Al and 1.7096 (12) Å for the Ga compound are slightly shorter than the average As—OH bond length in HAsO_4_^2−^ anions of 1.72 (3) Å (Schwendtner, 2008 ▸). The bonds to non-protonated O ligands are shorter than the average mean As—O bond length for inorganic arsenates (1.686 Å; Schwendtner, 2008 ▸), and fit well with the data for As bonds to non-protonated O atoms in H_1–3_AsO_4_ groups [average 1.669 Å, Schwendtner, 2008 ▸; 1.670 Å, AgAl(HAsO_4_)_2_; 1.675 Å, AgGa(HAsO_4_)_2_]. The average bond lengths for the AlO_6_ and GaO_6_ octa­hedra agree well with published averages (Baur, 1981 ▸; Overweg *et al.*, 1999 ▸). Bond-valence sums were calculated using the bond-valence parameters of Brown & Altermatt (1985 ▸), and amount to 0.88/0.90 (Ag1), 0.89 (Ag2), 3.05/3.16 (Al/Ga), 5.05/5.02 (As), 2.04/2.06 (O1), 1.85/1.85 (O2), 1.90/2.01 (O3) and 1.22/1.21 (O4 = OH; H atom not considered for calculation) valence units for AgAl(HAsO_4_)_2_ and AgGa(HAsO_4_)_2_, respectively. These results are reasonably close to the ideal values; the underbonded O2 is an acceptor of the strong hydrogen bond. Compared to isotypic KSc(HAsO_4_)_2_, the cell lengths and the hydrogen bonds of the two new compounds are considerably shorter. As a result of the similar ionic radii of Al^3+^ and Ga^3+^, these two compounds have similar unit-cell parameters. The Ga compound is slightly compressed along the *a* axis (smaller β), but elongated along the *b* axis; the *c* axis is not affected.

**Figure 3:**
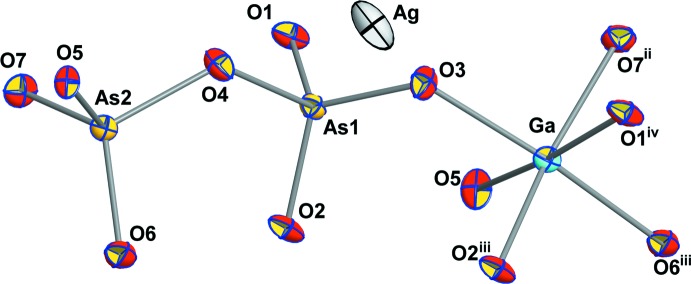
The principal building units of AgGaAs_2_O_7_ shown as displacement ellipsoids at the 70% probability level. [Symmetry codes: (ii) −*x*, *y* + 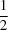, −*z* + 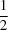; (iii) −*x*, −*y*, −*z*; (iv) *x*, −*y* − 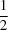, *z* − 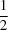.]

**Figure 4:**
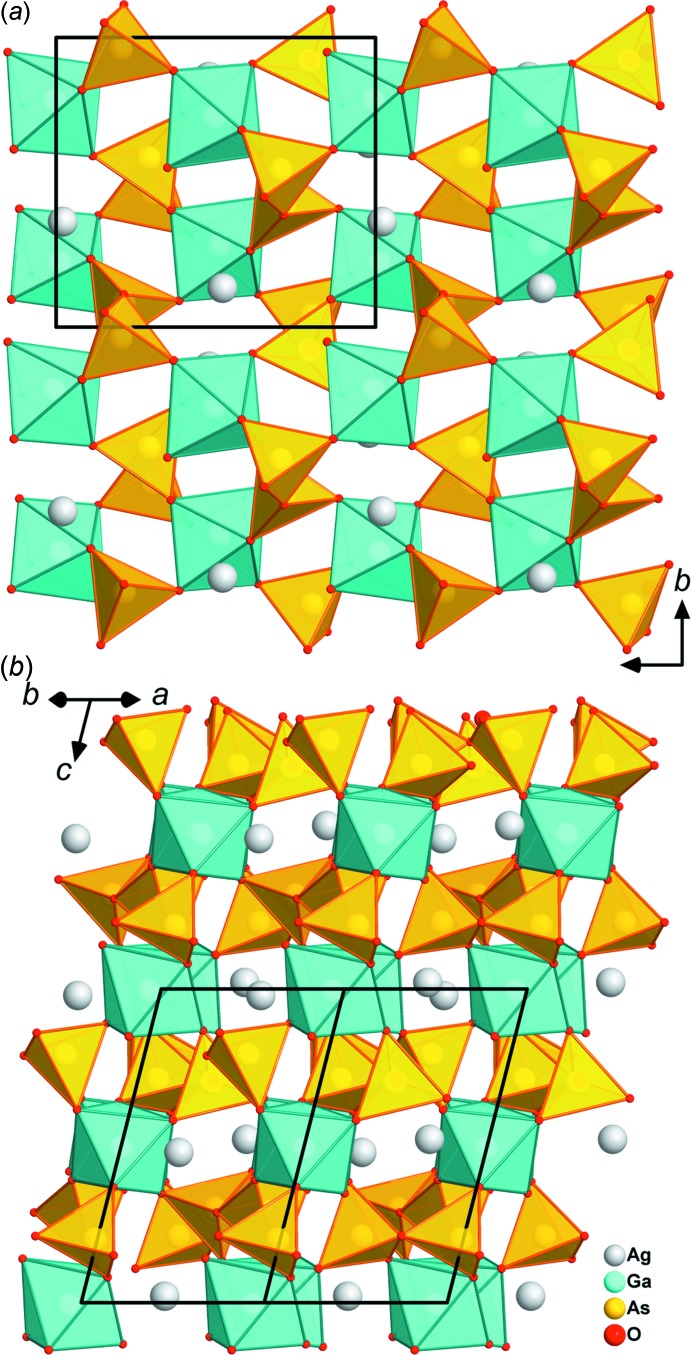
View of the framework structure of AgGaAs_2_O_7_ along (*a*) [100] and (*b*) [110].

The two diarsenates AgGaAs_2_O_7_ and NaGaAs_2_O_7_ crystallize in space group *P*2_1_/*c* and are isotypic to NaAlAs_2_O_7_ (Driss & Jouini, 1994 ▸). The asymmetric unit contains one Ag/Na, one Ga, two As and seven O sites, all of which occupy general positions (Fig. 3 ▸). The basic building block is an As_2_O_7_ group, which is connected to the GaO_6_ octa­hedra by two corners. The other four free O ligands are connected to different GaO_6_ octa­hedra to form a three-dimensional framework structure (Fig. 4 ▸). The As2—O_bridge_ distances [1.755 (3), 1.756 (3) Å] are nearly identical to the literature value of 1.755 Å for the average As—O_bridge_ bond length in diarsenates (Schwendtner & Kolitsch, 2007*d* ▸), whereas the As1—O_bridge_ distances [1.781 (3), 1.777 (3) Å] are further elongated. The average bond lengths of both AsO_4_ tetra­hedra are slightly longer than the literature value of 1.688 Å (Schwendtner & Kolitsch, 2007*d* ▸) for the average As—O bond length in As_2_O_7_ groups. The As—O—As angle is very close to the average value of As_2_O_7_ groups in a staggered conformation (124.2°, Schwendtner & Kolitsch, 2007*d* ▸) for both compounds (see Tables 5 ▸ and 6 ▸).

The GaO_6_ octa­hedra are slightly distorted and the average Ga—O bond lengths are close to the literature values (∼1.96 Å; Overweg *et al.*, 1999 ▸). The Na^+^ and Ag^+^ cations show a strongly distorted octa­hedral coordination. The calculated bond-valence sums using the parameters of Brown & Altermatt (1985 ▸) for Ag, Ga and As, and Wood & Palenik (1999 ▸) for Na, amount to 1.06/1.00 (Ag/Na), 3.11/3.11 (Ga), 4.90/4.93 (As1), 4.96/4.93 (As2), 2.08/2.11 (O1), 1.93/1.92 (O2), 2.01/2.01 (O3), 2.08/2.10 (O4), 1.87/1.86 (O5), 2.03/1.99 (O6), and 2.03/1.99 (O7) valence units for AgGaAs_2_O_7_ and NaGaAs_2_O_7_, respectively.

## Synthesis and crystallization   

The compounds were grown by hydro­thermal synthesis at 493 K (7 d, autogeneous pressure, slow furnace cooling) using Teflon-lined stainless-steel autoclaves with an approximate filling volume of 2 cm^3^. Reagent-grade Ag_2_CO_3_, Ga_2_O_3_, α-Al_2_O_3_, Na_2_CO_3_, and H_3_AsO_4_·0.5H_2_O were used as starting reagents in approximate volume ratios of *M* ^+^:*M* ^3+^:As of 1:1:2. The vessels were filled with distilled water to about 70% of their inner volumes, which led to final pH values of < 1 for all synthesis batches except NaGaAs_2_O_7_ (pH 1.5). The reaction products were washed thoroughly with distilled water, filtered and dried at room temperature.

**Figure 5:**
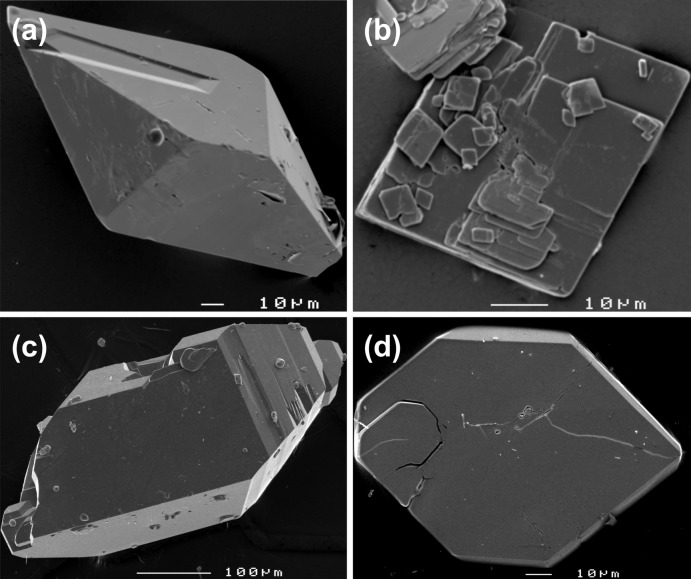
SEM micrographs of hydro­thermally synthesized crystals of (*a*) AgAl(HAsO_4_)_2_, (*b*) NaGaAs_2_O_7_, (*c*) AgGa(HAsO_4_)_2_ and (*d*) AgGaAs_2_O_7_.

The two hydrogen arsenates formed pseudo-dipyramidal colourless crystals up to 2 mm in length (yield > 95% for the Al compound with minor amounts of AgCl, explained by the reaction of Ag^+^ with remnants of concentrated hot HCl used for standard cleaning of the Teflon vessels before each new experiment; Fig. 5 ▸ *a*). The crystals of AgGa(HAsO_4_)_2_ (Fig. 5 ▸ *c*) were accompanied by about 20% of AgGaAs_2_O_7_ as pseudo-ortho­rhom­bic platelets (Fig. 5 ▸ *d*). NaGaAs_2_O_7_ crystallized as small, colourless platelets with a diamond-shaped outline (yield 100%) (Fig. 5 ▸ *b*).

Measured X-ray powder diffraction diagrams of the NaGaAs_2_O_7_ and AgAl(HAsO_4_)_2_ synthesis batches were deposited at the Inter­national Centre for Diffraction Data under PDF number 57-0162 (Prem *et al.*, 2005 ▸) for NaGaAs_2_O_7_ and 57-0161 (Prem *et al.*, 2005 ▸) for AgAl(HAsO_4_)_2_.

## Refinement   

Crystal data, data collection and structure refinement details are summarized in Table 7 ▸.

For easier comparison, the atomic positions of the isotypic compounds KSc(HAsO_4_)_2_ (Schwendtner & Kolitsch, 2004 ▸) and NaAlAs_2_O_7_ (Driss & Jouini, 1994 ▸) were used for the final refinement. The O—H distance was restrained to 0.9 (2) Å for AgAl(HAsO_4_)_2_ and refined freely for AgGa(HAsO_4_)_2_.

Remaining electron densities are below 1 e Å^−3^ for the two hydrogen arsenates. The remaining maximum and minimum electron densities in the final difference-Fourier maps are located close to the As1 atom (0.87/0.78 Å) for NaGaAs_2_O_7_, and close to the Ag atom (0.73/0.66 Å) for AgGaAs_2_O_7_.

## Supplementary Material

Crystal structure: contains datablock(s) AgAlHAsO42, AgGaHAsO42, AgGaAs2O7, NaGaAs2O7. DOI: 10.1107/S2056989017005631/pk2600sup1.cif

Structure factors: contains datablock(s) AgAlHAsO42. DOI: 10.1107/S2056989017005631/pk2600AgAlHAsO42sup2.hkl

Structure factors: contains datablock(s) AgGaHAsO42. DOI: 10.1107/S2056989017005631/pk2600AgGaHAsO42sup3.hkl

Structure factors: contains datablock(s) AgGaAs2O7. DOI: 10.1107/S2056989017005631/pk2600AgGaAs2O7sup4.hkl

Structure factors: contains datablock(s) NaGaAs2O7. DOI: 10.1107/S2056989017005631/pk2600NaGaAs2O7sup5.hkl

Supporting information file. DOI: 10.1107/S2056989017005631/pk2600NaGaAs2O7sup6.cml

CCDC references: 1543927, 1543926, 1543925, 1543924

Additional supporting information:  crystallographic information; 3D view; checkCIF report

## Figures and Tables

**Table 1:** Selected bond lengths (Å) for AgAl(HAsO_4_)_2_

Ag1—O1	2.4040 (11)	Al—O3^iv^	1.9167 (10)
Ag1—O3^i^	2.6362 (11)	As—O2	1.6683 (9)
Ag1—O4^ii^	2.8202 (13)	As—O1	1.6697 (10)
Ag1—O2	3.1259 (11)	As—O3	1.6710 (11)
Al—O1	1.8920 (10)	As—O4	1.7161 (10)
Al—O2^iii^	1.8955 (11)		

Symmetry codes: (i) 
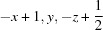
; (ii) 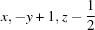; (iii) 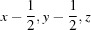; (iv) 
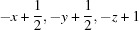
.

**Table 2:** Hydrogen-bond geometry (Å, °) for AgAl(HAsO_4_)_2_

*D*—H⋯*A*	*D*—H	H⋯*A*	*D*⋯*A*	*D*—H⋯*A*
O4—H⋯O2^v^	0.89 (1)	1.81 (2)	2.6212 (17)	151 (3)

Symmetry code: (v) 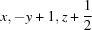.

**Table 3:** Selected bond lengths (Å) for AgGa(HAsO_4_)_2_

Ag1—O1	2.3600 (12)	Ag2—O2^iii^	3.116 (18)
Ag1—O3^i^	2.5864 (12)	Ag2—O2	3.185 (17)
Ag1—O4^ii^	3.0574 (15)	Ga—O1	1.9591 (11)
Ag1—O2	3.1352 (12)	Ga—O2^vi^	1.9641 (11)
Ag2—O1	2.357 (17)	Ga—O3^vii^	1.9799 (12)
Ag2—O1^iii^	2.402 (18)	As—O2	1.6719 (11)
Ag2—O3^i^	2.48 (2)	As—O3	1.6753 (12)
Ag2—O3^iv^	2.73 (3)	As—O1	1.6773 (11)
Ag2—O4^v^	2.83 (2)	As—O4	1.7096 (12)

Symmetry codes: (i) 
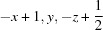
; (ii) 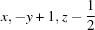; (iii) 
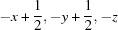
; (iv) 
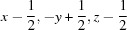
; (v) 
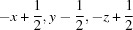
; (vi) 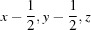; (vii) 
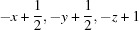
.

**Table 4:** Hydrogen-bond geometry (Å, °) for AgGa(HAsO_4_)_2_

*D*—H⋯*A*	*D*—H	H⋯*A*	*D*⋯*A*	*D*—H⋯*A*
O4—H⋯O2^viii^	0.80 (3)	1.89 (3)	2.6240 (19)	153 (3)

Symmetry code: (viii) 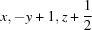.

**Table 5:** Selected geometric parameters (Å, °) for AgGa(As_2_O_7_)

Ag—O1^i^	2.353 (3)	Ga—O6^v^	1.985 (3)
Ag—O6^ii^	2.361 (3)	Ga—O7^i^	2.015 (3)
Ag—O3	2.440 (3)	As1—O2	1.653 (3)
Ag—O7^iii^	2.529 (3)	As1—O1	1.668 (3)
Ag—O7^iv^	2.600 (3)	As1—O3	1.679 (3)
Ag—O4	2.766 (3)	As1—O4	1.781 (3)
Ga—O2^v^	1.939 (3)	As2—O5^vi^	1.655 (3)
Ga—O3	1.957 (3)	As2—O7	1.674 (3)
Ga—O1^iii^	1.973 (3)	As2—O6	1.675 (3)
Ga—O5	1.975 (3)	As2—O4	1.755 (3)
			
As2—O4—As1	124.65 (15)		

Symmetry codes: (i) 
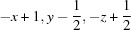
; (ii) 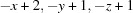; (iii) 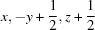; (iv) 
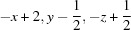
; (v) 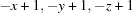; (vi) 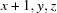.

**Table 6:** Selected geometric parameters (Å, °) for NaGa(As_2_O_7_)

Na—O1^i^	2.289 (4)	Ga—O6^v^	1.987 (3)
Na—O6^ii^	2.360 (4)	Ga—O7^i^	2.042 (3)
Na—O3	2.364 (4)	As1—O2	1.655 (3)
Na—O7^iii^	2.468 (4)	As1—O1	1.664 (3)
Na—O7^iv^	2.479 (4)	As1—O3	1.676 (3)
Na—O4	2.624 (4)	As1—O4	1.777 (3)
Ga—O2^v^	1.940 (3)	As2—O5^vi^	1.661 (3)
Ga—O1^iii^	1.952 (3)	As2—O6	1.671 (3)
Ga—O3	1.960 (3)	As2—O7	1.678 (3)
Ga—O5	1.967 (3)	As2—O4	1.756 (3)
			
As2—O4—As1	124.73 (19)		

Symmetry codes: (i) 
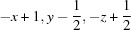
; (ii) 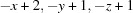; (iii) 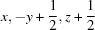; (iv) 
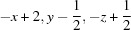
; (v) 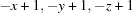; (vi) 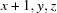.

**Table 7:** Experimental details

	AgAl(HAsO_4_)_2_	AgGa(HAsO_4_)_2_	AgGa(AsO_7_)	NaGa(As_2_O_7_)
Crystal data				
*M*_r_	414.71	457.45	354.55	439.43
Crystal system, space group	Monoclinic, *C*2/*c*	Monoclinic, *C*2/*c*	Monoclinic, *P*2_1_/*c*	Monoclinic, *P*2_1_/*c*
Temperature (K)	293	293	293	293
*a*, *b*, *c* (Å)	7.842 (2), 9.937 (2), 8.686 (2)	7.826 (2), 10.216 (2), 8.694 (2)	6.987 (1), 8.266 (2), 9.677 (2)	7.049 (1), 8.368 (2), 9.735 (2)
β (°)	108.45 (3)	107.77 (3)	107.50 (3)	108.47 (3)
*V* (Å^3^)	642.1 (3)	661.9 (3)	533.0 (2)	544.7 (2)
*Z*	4	4	4	4
Radiation type	Mo *K*α	Mo *K*α	Mo *K*α	Mo *K*α
μ (mm^−1^)	13.51	16.96	17.55	20.58
Crystal size (mm)	0.15 × 0.08 × 0.07	0.18 × 0.10 × 0.10	0.07 × 0.07 × 0.02	0.10 × 0.08 × 0.02

Data collection				
Diffractometer	Nonius KappaCCD single-crystal four-circle	Nonius KappaCCD single-crystal four-circle	Nonius KappaCCD single-crystal four-circle	Nonius KappaCCD single-crystal four-circle
Absorption correction	Multi-scan (*HKL* *SCALEPACK*; Otwinowski *et al.*, 2003 ▸)	Multi-scan (*HKL* *SCALEPACK*; Otwinowski *et al.*, 2003 ▸)	Multi-scan (*HKL* *SCALEPACK*; Otwinowski *et al.*, 2003 ▸)	Multi-scan (*HKL* *SCALEPACK*; Otwinowski *et al.*, 2003 ▸)
*T*_min_, *T*_max_	0.236, 0.451	0.150, 0.282	0.373, 0.720	0.233, 0.684
No. of measured, independent and observed [*I* > 2σ(*I*)] reflections	2751, 1408, 1354	2852, 1460, 1402	3015, 1557, 1336	3849, 1982, 1775
*R*_int_	0.011	0.012	0.023	0.022
(sin θ/λ)_max_ (Å^−1^)	0.806	0.806	0.704	0.757

Refinement				
*R*[*F* ^2^ > 2σ(*F* ^2^)], *wR*(*F* ^2^), *S*	0.014, 0.037, 1.10	0.015, 0.037, 1.15	0.033, 0.090, 1.04	0.033, 0.089, 1.05
No. of reflections	1408	1460	1557	1982
No. of parameters	62	73	100	100
No. of restraints	1	0	0	0
H-atom treatment	H atoms treated by a mixture of independent and constrained refinement	All H-atom parameters refined	–	–
Δρ_max_, Δρ_min_ (e Å^−3^)	0.55, −0.60	0.66, −0.50	1.54, −1.49	2.29, −2.38

Computer programs: *COLLECT* (Nonius, 2003 ▸), *HKL* *DENZO* and *SCALEPACK* (Otwinowski *et al.*, 2003 ▸), *SHELXS97* (Sheldrick, 2008 ▸), *SHELXL2016* (Sheldrick, 2015 ▸), *DIAMOND* (Brandenburg, 2005 ▸) and *publCIF* (Westrip, 2010 ▸).

## supplementary crystallographic information

### (AgAlHAsO42) Silver(I) aluminium bis[hydrogen arsenate(V)] Crystal data

**Table:**

AgAl(HAsO_4_)_2_	*F*(000) = 768
*M**_r_* = 414.71	*D*_x_ = 4.290 Mg m^−^^3^
Monoclinic, *C*2/*c*	Mo *K*α radiation, λ = 0.71073 Å
*a* = 7.842 (2) Å	Cell parameters from 1477 reflections
*b* = 9.937 (2) Å	θ = 2.0–35.0°
*c* = 8.686 (2) Å	µ = 13.51 mm^−^^1^
β = 108.45 (3)°	*T* = 293 K
*V* = 642.1 (3) Å^3^	Pseudo-dipyramidal glassy, colourless
*Z* = 4	0.15 × 0.08 × 0.07 mm

### (AgAlHAsO42) Silver(I) aluminium bis[hydrogen arsenate(V)] Data collection

**Table:**

Nonius KappaCCD single-crystal four-circle diffractometer	1354 reflections with *I* > 2σ(*I*)
Radiation source: fine-focus sealed tube	*R*_int_ = 0.011
φ and ω scans	θ_max_ = 34.9°, θ_min_ = 3.4°
Absorption correction: multi-scan (HKL SCALEPACK; Otwinowski *et al.*, 2003)	*h* = −12→12
*T*_min_ = 0.236, *T*_max_ = 0.451	*k* = −16→16
2751 measured reflections	*l* = −13→13
1408 independent reflections	

### (AgAlHAsO42) Silver(I) aluminium bis[hydrogen arsenate(V)] Refinement

**Table:**

Refinement on *F*^2^	Secondary atom site location: difference Fourier map
Least-squares matrix: full	Hydrogen site location: difference Fourier map
*R*[*F*^2^ > 2σ(*F*^2^)] = 0.014	H atoms treated by a mixture of independent and constrained refinement
*wR*(*F*^2^) = 0.037	*w* = 1/[σ^2^(*F*_o_^2^) + (0.018*P*)^2^ + 0.890*P*] where *P* = (*F*_o_^2^ + 2*F*_c_^2^)/3
*S* = 1.10	(Δ/σ)_max_ = 0.001
1408 reflections	Δρ_max_ = 0.55 e Å^−^^3^
62 parameters	Δρ_min_ = −0.60 e Å^−^^3^
1 restraint	Extinction correction: SHELXL2016 (Sheldrick, 2015), Fc^*^=kFc[1+0.001xFc^2^λ^3^/sin(2θ)]^-1/4^
Primary atom site location: structure-invariant direct methods	Extinction coefficient: 0.0114 (3)

### (AgAlHAsO42) Silver(I) aluminium bis[hydrogen arsenate(V)] Special details

**Table:**

Geometry. All esds (except the esd in the dihedral angle between two l.s. planes) are estimated using the full covariance matrix. The cell esds are taken into account individually in the estimation of esds in distances, angles and torsion angles; correlations between esds in cell parameters are only used when they are defined by crystal symmetry. An approximate (isotropic) treatment of cell esds is used for estimating esds involving l.s. planes.

### (AgAlHAsO42) Silver(I) aluminium bis[hydrogen arsenate(V)] Fractional atomic coordinates and isotropic or equivalent isotropic displacement parameters (Å^2^)

**Table:**

	*x*	*y*	*z*	*U*_iso_*/*U*_eq_	
Ag1	0.250000	0.250000	0.000000	0.03518 (7)	
Al	0.000000	0.13916 (5)	0.250000	0.00554 (9)	
As	0.27443 (2)	0.40001 (2)	0.35945 (2)	0.00508 (5)	
O1	0.17964 (13)	0.26607 (10)	0.24940 (12)	0.00972 (16)	
O2	0.32668 (13)	0.50141 (10)	0.22800 (11)	0.00973 (16)	
O3	0.44856 (12)	0.35479 (10)	0.51919 (11)	0.00870 (15)	
O4	0.12010 (13)	0.48544 (12)	0.42496 (12)	0.01492 (19)	
H	0.153 (4)	0.487 (3)	0.5324 (12)	0.047 (9)*	

### (AgAlHAsO42) Silver(I) aluminium bis[hydrogen arsenate(V)] Atomic displacement parameters (Å^2^)

**Table:**

	*U*^11^	*U*^22^	*U*^33^	*U*^12^	*U*^13^	*U*^23^
Ag1	0.06317 (16)	0.02460 (10)	0.03540 (12)	−0.01037 (9)	0.04068 (11)	−0.00702 (8)
Al	0.0059 (2)	0.0053 (2)	0.0052 (2)	0.000	0.00145 (16)	0.000
As	0.00555 (6)	0.00526 (7)	0.00416 (6)	−0.00071 (3)	0.00115 (4)	0.00007 (3)
O1	0.0112 (4)	0.0086 (4)	0.0102 (4)	−0.0057 (3)	0.0045 (3)	−0.0046 (3)
O2	0.0113 (4)	0.0100 (4)	0.0069 (3)	−0.0043 (3)	0.0014 (3)	0.0027 (3)
O3	0.0087 (3)	0.0117 (4)	0.0045 (3)	0.0024 (3)	0.0004 (3)	0.0009 (3)
O4	0.0113 (4)	0.0234 (5)	0.0104 (4)	0.0070 (4)	0.0038 (3)	−0.0026 (4)

### (AgAlHAsO42) Silver(I) aluminium bis[hydrogen arsenate(V)] Geometric parameters (Å, º)

**Table:**

Ag1—O1^i^	2.4040 (11)	Al—O2^vii^	1.8955 (11)
Ag1—O1	2.4040 (11)	Al—O2^v^	1.8955 (11)
Ag1—O3^ii^	2.6362 (11)	Al—O3^viii^	1.9167 (10)
Ag1—O3^iii^	2.6362 (11)	Al—O3^iii^	1.9167 (10)
Ag1—O4^iv^	2.8202 (13)	As—O2	1.6683 (9)
Ag1—O4^v^	2.8202 (13)	As—O1	1.6697 (10)
Ag1—O2	3.1259 (11)	As—O3	1.6710 (11)
Ag1—O2^i^	3.1259 (11)	As—O4	1.7161 (10)
Al—O1	1.8920 (10)	O4—H	0.886 (10)
Al—O1^vi^	1.8920 (10)		
			
O1—Al—O1^vi^	96.40 (7)	O1^vi^—Al—O3^iii^	94.10 (5)
O1—Al—O2^vii^	172.66 (4)	O2^vii^—Al—O3^iii^	90.59 (5)
O1^vi^—Al—O2^vii^	88.33 (5)	O2^v^—Al—O3^iii^	92.00 (5)
O1—Al—O2^v^	88.33 (5)	O3^viii^—Al—O3^iii^	176.41 (7)
O1^vi^—Al—O2^v^	172.66 (4)	O2—As—O1	104.53 (5)
O2^vii^—Al—O2^v^	87.54 (7)	O2—As—O3	114.68 (5)
O1—Al—O3^viii^	94.10 (5)	O1—As—O3	111.09 (5)
O1^vi^—Al—O3^viii^	83.49 (5)	O2—As—O4	106.25 (6)
O2^vii^—Al—O3^viii^	92.00 (5)	O1—As—O4	110.56 (5)
O2^v^—Al—O3^viii^	90.59 (5)	O3—As—O4	109.54 (5)
O1—Al—O3^iii^	83.49 (5)		

Symmetry codes: (i) −*x*+1/2, −*y*+1/2, −*z*; (ii) −*x*+1, *y*, −*z*+1/2; (iii) *x*−1/2, −*y*+1/2, *z*−1/2; (iv) *x*, −*y*+1, *z*−1/2; (v) −*x*+1/2, *y*−1/2, −*z*+1/2; (vi) −*x*, *y*, −*z*+1/2; (vii) *x*−1/2, *y*−1/2, *z*; (viii) −*x*+1/2, −*y*+1/2, −*z*+1.

### (AgAlHAsO42) Silver(I) aluminium bis[hydrogen arsenate(V)] Hydrogen-bond geometry (Å, º)

**Table:**

*D*—H···*A*	*D*—H	H···*A*	*D*···*A*	*D*—H···*A*
O4—H···O2^ix^	0.89 (1)	1.81 (2)	2.6212 (17)	151 (3)

Symmetry code: (ix) *x*, −*y*+1, *z*+1/2.

### (AgGaHAsO42) Silver(I) gallium bis[hydrogen arsenate(V)] Crystal data

**Table:**

AgGa(HAsO_4_)_2_	*F*(000) = 840
*M**_r_* = 457.45	*D*_x_ = 4.590 Mg m^−^^3^
Monoclinic, *C*2/*c*	Mo *K*α radiation, λ = 0.71073 Å
*a* = 7.826 (2) Å	Cell parameters from 1519 reflections
*b* = 10.216 (2) Å	θ = 2.0–35.0°
*c* = 8.694 (2) Å	µ = 16.96 mm^−^^1^
β = 107.77 (3)°	*T* = 293 K
*V* = 661.9 (3) Å^3^	Large pseudo-dipyramidal glassy, colourless
*Z* = 4	0.18 × 0.10 × 0.10 mm

### (AgGaHAsO42) Silver(I) gallium bis[hydrogen arsenate(V)] Data collection

**Table:**

Nonius KappaCCD single-crystal four-circle diffractometer	1402 reflections with *I* > 2σ(*I*)
Radiation source: fine-focus sealed tube	*R*_int_ = 0.012
φ and ω scans	θ_max_ = 35.0°, θ_min_ = 3.4°
Absorption correction: multi-scan (HKL SCALEPACK; Otwinowski *et al.*, 2003)	*h* = −12→12
*T*_min_ = 0.150, *T*_max_ = 0.282	*k* = −16→16
2852 measured reflections	*l* = −14→13
1460 independent reflections	

### (AgGaHAsO42) Silver(I) gallium bis[hydrogen arsenate(V)] Refinement

**Table:**

Refinement on *F*^2^	Secondary atom site location: difference Fourier map
Least-squares matrix: full	Hydrogen site location: difference Fourier map
*R*[*F*^2^ > 2σ(*F*^2^)] = 0.015	All H-atom parameters refined
*wR*(*F*^2^) = 0.037	*w* = 1/[σ^2^(*F*_o_^2^) + (0.015*P*)^2^ + 0.940*P*] where *P* = (*F*_o_^2^ + 2*F*_c_^2^)/3
*S* = 1.15	(Δ/σ)_max_ = 0.037
1460 reflections	Δρ_max_ = 0.66 e Å^−^^3^
73 parameters	Δρ_min_ = −0.50 e Å^−^^3^
0 restraints	Extinction correction: SHELXL2016 (Sheldrick, 2015), Fc^*^=kFc[1+0.001xFc^2^λ^3^/sin(2θ)]^-1/4^
Primary atom site location: structure-invariant direct methods	Extinction coefficient: 0.0133 (3)

### (AgGaHAsO42) Silver(I) gallium bis[hydrogen arsenate(V)] Special details

**Table:**

Geometry. All esds (except the esd in the dihedral angle between two l.s. planes) are estimated using the full covariance matrix. The cell esds are taken into account individually in the estimation of esds in distances, angles and torsion angles; correlations between esds in cell parameters are only used when they are defined by crystal symmetry. An approximate (isotropic) treatment of cell esds is used for estimating esds involving l.s. planes.

### (AgGaHAsO42) Silver(I) gallium bis[hydrogen arsenate(V)] Fractional atomic coordinates and isotropic or equivalent isotropic displacement parameters (Å^2^)

**Table:**

	*x*	*y*	*z*	*U*_iso_*/*U*_eq_	Occ. (<1)
Ag1	0.250000	0.250000	0.000000	0.0335 (9)	0.75 (2)
Ag2	0.284 (3)	0.2351 (14)	0.019 (2)	0.0316 (18)	0.123 (10)
Ga	0.000000	0.13670 (2)	0.250000	0.00500 (5)	
As	0.27567 (2)	0.39556 (2)	0.35733 (2)	0.00482 (5)	
O1	0.18942 (15)	0.26190 (10)	0.24943 (13)	0.00983 (18)	
O2	0.32045 (15)	0.49738 (11)	0.22405 (13)	0.01055 (19)	
O3	0.45491 (14)	0.35629 (11)	0.51333 (13)	0.00948 (18)	
O4	0.11608 (16)	0.46896 (14)	0.42534 (15)	0.0169 (2)	
H	0.148 (4)	0.487 (3)	0.519 (4)	0.033 (8)*	

### (AgGaHAsO42) Silver(I) gallium bis[hydrogen arsenate(V)] Atomic displacement parameters (Å^2^)

**Table:**

	*U*^11^	*U*^22^	*U*^33^	*U*^12^	*U*^13^	*U*^23^
Ag1	0.051 (2)	0.0368 (14)	0.0271 (9)	−0.0173 (12)	0.0327 (12)	−0.0125 (9)
Ag2	0.049 (4)	0.0249 (16)	0.035 (3)	−0.015 (2)	0.034 (3)	−0.0064 (14)
Ga	0.00555 (9)	0.00478 (9)	0.00482 (9)	0.000	0.00182 (6)	0.000
As	0.00564 (7)	0.00525 (7)	0.00364 (7)	−0.00073 (4)	0.00153 (5)	−0.00003 (4)
O1	0.0114 (4)	0.0093 (4)	0.0101 (5)	−0.0057 (3)	0.0053 (4)	−0.0045 (3)
O2	0.0122 (4)	0.0110 (4)	0.0068 (4)	−0.0060 (3)	0.0003 (3)	0.0036 (4)
O3	0.0085 (4)	0.0135 (4)	0.0053 (4)	0.0032 (3)	0.0004 (3)	0.0011 (4)
O4	0.0116 (5)	0.0299 (6)	0.0094 (5)	0.0081 (4)	0.0035 (4)	−0.0047 (4)

### (AgGaHAsO42) Silver(I) gallium bis[hydrogen arsenate(V)] Geometric parameters (Å, º)

**Table:**

Ag1—O1^i^	2.3600 (12)	Ag2—O2	3.185 (17)
Ag1—O1	2.3600 (12)	Ga—O1	1.9591 (11)
Ag1—O3^ii^	2.5864 (12)	Ga—O1^vi^	1.9591 (11)
Ag1—O3^iii^	2.5864 (12)	Ga—O2^vii^	1.9641 (11)
Ag1—O4^iv^	3.0574 (15)	Ga—O2^v^	1.9641 (11)
Ag1—O4^v^	3.0574 (15)	Ga—O3^viii^	1.9799 (12)
Ag1—O2	3.1352 (12)	Ga—O3^iii^	1.9799 (12)
Ag2—O1	2.357 (17)	As—O2	1.6719 (11)
Ag2—O1^i^	2.402 (18)	As—O3	1.6753 (12)
Ag2—O3^ii^	2.48 (2)	As—O1	1.6773 (11)
Ag2—O3^iii^	2.73 (3)	As—O4	1.7096 (12)
Ag2—O4^v^	2.83 (2)	O4—H	0.80 (3)
Ag2—O2^i^	3.116 (18)		
			
O1—Ga—O1^vi^	98.48 (7)	O3^viii^—Ga—O3^iii^	175.86 (6)
O1—Ga—O2^vii^	171.19 (5)	O2—As—O3	114.16 (6)
O1^vi^—Ga—O2^vii^	87.59 (5)	O2—As—O1	104.63 (6)
O1—Ga—O2^v^	87.59 (5)	O3—As—O1	110.64 (6)
O1^vi^—Ga—O2^v^	171.19 (5)	O2—As—O4	107.25 (7)
O2^vii^—Ga—O2^v^	87.12 (7)	O3—As—O4	110.16 (6)
O1—Ga—O3^viii^	94.82 (5)	O1—As—O4	109.80 (6)
O1^vi^—Ga—O3^viii^	82.46 (5)	O2—As—O3	114.16 (6)
O2^vii^—Ga—O3^viii^	92.29 (5)	O2—As—O1	104.63 (6)
O2^v^—Ga—O3^viii^	90.71 (5)	O3—As—O1	110.64 (6)
O1—Ga—O3^iii^	82.46 (5)	O2—As—O4	107.25 (7)
O1^vi^—Ga—O3^iii^	94.82 (5)	O3—As—O4	110.16 (6)
O2^vii^—Ga—O3^iii^	90.71 (5)	O1—As—O4	109.80 (6)
O2^v^—Ga—O3^iii^	92.29 (5)		

Symmetry codes: (i) −*x*+1/2, −*y*+1/2, −*z*; (ii) −*x*+1, *y*, −*z*+1/2; (iii) *x*−1/2, −*y*+1/2, *z*−1/2; (iv) *x*, −*y*+1, *z*−1/2; (v) −*x*+1/2, *y*−1/2, −*z*+1/2; (vi) −*x*, *y*, −*z*+1/2; (vii) *x*−1/2, *y*−1/2, *z*; (viii) −*x*+1/2, −*y*+1/2, −*z*+1.

### (AgGaHAsO42) Silver(I) gallium bis[hydrogen arsenate(V)] Hydrogen-bond geometry (Å, º)

**Table:**

*D*—H···*A*	*D*—H	H···*A*	*D*···*A*	*D*—H···*A*
O4—H···O2^ix^	0.80 (3)	1.89 (3)	2.6240 (19)	153 (3)

Symmetry code: (ix) *x*, −*y*+1, *z*+1/2.

### (AgGaAs2O7) Silver(I) gallium diarsenate(V) Crystal data

**Table:**

AgGa(As_2_O_7_)	*F*(000) = 800
*M**_r_* = 439.43	*D*_x_ = 5.359 Mg m^−^^3^
Monoclinic, *P*2_1_/*c*	Mo *K*α radiation, λ = 0.71073 Å
*a* = 7.049 (1) Å	Cell parameters from 2105 reflections
*b* = 8.368 (2) Å	θ = 2.0–32.6°
*c* = 9.735 (2) Å	µ = 20.58 mm^−^^1^
β = 108.47 (3)°	*T* = 293 K
*V* = 544.7 (2) Å^3^	Pseudo-orthorhombic platelets, colourless
*Z* = 4	0.10 × 0.08 × 0.02 mm

### (AgGaAs2O7) Silver(I) gallium diarsenate(V) Data collection

**Table:**

Nonius KappaCCD single-crystal four-circle diffractometer	1775 reflections with *I* > 2σ(*I*)
Radiation source: fine-focus sealed tube	*R*_int_ = 0.022
φ and ω scans	θ_max_ = 32.5°, θ_min_ = 3.1°
Absorption correction: multi-scan (HKL SCALEPACK; Otwinowski *et al.*, 2003)	*h* = −10→10
*T*_min_ = 0.233, *T*_max_ = 0.684	*k* = −12→12
3849 measured reflections	*l* = −14→14
1982 independent reflections	

### (AgGaAs2O7) Silver(I) gallium diarsenate(V) Refinement

**Table:**

Refinement on *F*^2^	0 restraints
Least-squares matrix: full	Primary atom site location: structure-invariant direct methods
*R*[*F*^2^ > 2σ(*F*^2^)] = 0.033	Secondary atom site location: difference Fourier map
*wR*(*F*^2^) = 0.089	*w* = 1/[σ^2^(*F*_o_^2^) + (0.0619*P*)^2^ + 0.6339*P*] where *P* = (*F*_o_^2^ + 2*F*_c_^2^)/3
*S* = 1.05	(Δ/σ)_max_ < 0.001
1982 reflections	Δρ_max_ = 2.29 e Å^−^^3^
100 parameters	Δρ_min_ = −2.38 e Å^−^^3^

### (AgGaAs2O7) Silver(I) gallium diarsenate(V) Special details

**Table:**

Geometry. All esds (except the esd in the dihedral angle between two l.s. planes) are estimated using the full covariance matrix. The cell esds are taken into account individually in the estimation of esds in distances, angles and torsion angles; correlations between esds in cell parameters are only used when they are defined by crystal symmetry. An approximate (isotropic) treatment of cell esds is used for estimating esds involving l.s. planes.

### (AgGaAs2O7) Silver(I) gallium diarsenate(V) Fractional atomic coordinates and isotropic or equivalent isotropic displacement parameters (Å^2^)

**Table:**

	*x*	*y*	*z*	*U*_iso_*/*U*_eq_	
Ag	0.81152 (5)	0.13552 (4)	0.47918 (4)	0.02028 (11)	
Ga	0.28442 (6)	0.28038 (5)	0.49133 (4)	0.00810 (11)	
As1	0.52020 (5)	0.41622 (4)	0.28916 (4)	0.00783 (10)	
As2	0.94310 (5)	0.54112 (4)	0.29927 (4)	0.00759 (10)	
O1	0.3828 (4)	0.4083 (3)	0.1148 (3)	0.0108 (5)	
O2	0.5231 (4)	0.5961 (3)	0.3601 (3)	0.0118 (5)	
O3	0.4848 (4)	0.2646 (3)	0.3914 (3)	0.0107 (5)	
O4	0.7697 (4)	0.3871 (3)	0.2876 (3)	0.0118 (5)	
O5	0.1621 (4)	0.4491 (3)	0.3489 (3)	0.0117 (5)	
O6	0.9278 (4)	0.6807 (3)	0.4187 (3)	0.0126 (5)	
O7	0.8992 (4)	0.6146 (3)	0.1321 (3)	0.0113 (5)	

### (AgGaAs2O7) Silver(I) gallium diarsenate(V) Atomic displacement parameters (Å^2^)

**Table:**

	*U*^11^	*U*^22^	*U*^33^	*U*^12^	*U*^13^	*U*^23^
Ag	0.01549 (17)	0.01302 (15)	0.0269 (2)	−0.00362 (10)	−0.00097 (13)	0.00458 (11)
Ga	0.0088 (2)	0.00743 (18)	0.00749 (19)	0.00004 (12)	0.00177 (15)	0.00004 (12)
As1	0.00867 (19)	0.00705 (17)	0.00701 (17)	−0.00073 (11)	0.00144 (13)	−0.00004 (11)
As2	0.00788 (18)	0.00726 (17)	0.00707 (18)	0.00035 (11)	0.00158 (13)	0.00016 (11)
O1	0.0143 (13)	0.0076 (11)	0.0076 (11)	0.0007 (9)	−0.0008 (10)	0.0002 (8)
O2	0.0133 (13)	0.0080 (11)	0.0113 (12)	−0.0024 (9)	0.0002 (10)	−0.0026 (9)
O3	0.0125 (12)	0.0096 (11)	0.0114 (11)	0.0030 (9)	0.0056 (10)	0.0040 (9)
O4	0.0104 (12)	0.0080 (11)	0.0164 (13)	−0.0007 (9)	0.0033 (10)	0.0003 (9)
O5	0.0075 (11)	0.0128 (11)	0.0144 (13)	0.0043 (9)	0.0030 (10)	0.0060 (10)
O6	0.0122 (13)	0.0141 (12)	0.0120 (12)	−0.0042 (10)	0.0043 (10)	−0.0052 (10)
O7	0.0129 (13)	0.0124 (12)	0.0074 (11)	0.0015 (9)	0.0013 (10)	0.0020 (9)

### (AgGaAs2O7) Silver(I) gallium diarsenate(V) Geometric parameters (Å, º)

**Table:**

Ag—O1^i^	2.353 (3)	Ga—O6^v^	1.985 (3)
Ag—O6^ii^	2.361 (3)	Ga—O7^i^	2.015 (3)
Ag—O3	2.440 (3)	As1—O2	1.653 (3)
Ag—O7^iii^	2.529 (3)	As1—O1	1.668 (3)
Ag—O7^iv^	2.600 (3)	As1—O3	1.679 (3)
Ag—O4	2.766 (3)	As1—O4	1.781 (3)
Ga—O2^v^	1.939 (3)	As2—O5^vi^	1.655 (3)
Ga—O3	1.957 (3)	As2—O7	1.674 (3)
Ga—O1^iii^	1.973 (3)	As2—O6	1.675 (3)
Ga—O5	1.975 (3)	As2—O4	1.755 (3)
			
O1^i^—Ag—O6^ii^	165.93 (10)	O3—Ga—O7^i^	94.90 (12)
O1^i^—Ag—O3	81.53 (10)	O1^iii^—Ga—O7^i^	81.24 (11)
O6^ii^—Ag—O3	112.42 (10)	O5—Ga—O7^i^	91.08 (12)
O1^i^—Ag—O7^iii^	64.14 (9)	O6^v^—Ga—O7^i^	86.83 (11)
O6^ii^—Ag—O7^iii^	106.19 (10)	O2—As1—O1	112.81 (13)
O3—Ag—O7^iii^	126.87 (9)	O2—As1—O3	115.14 (14)
O2^v^—Ga—O3	87.83 (12)	O1—As1—O3	115.19 (13)
O2^v^—Ga—O1^iii^	86.80 (11)	O2—As1—O4	104.27 (13)
O3—Ga—O1^iii^	94.54 (11)	O1—As1—O4	104.04 (14)
O2^v^—Ga—O5	100.89 (12)	O3—As1—O4	103.55 (13)
O3—Ga—O5	85.56 (11)	O5^vi^—As2—O7	108.84 (14)
O1^iii^—Ga—O5	172.30 (11)	O5^vi^—As2—O6	112.38 (15)
O2^v^—Ga—O6^v^	91.74 (12)	O7—As2—O6	112.71 (14)
O3—Ga—O6^v^	173.63 (11)	O5^vi^—As2—O4	104.10 (13)
O1^iii^—Ga—O6^v^	91.78 (12)	O7—As2—O4	107.22 (13)
O5—Ga—O6^v^	88.28 (11)	O6—As2—O4	111.12 (14)
O2^v^—Ga—O7^i^	167.90 (11)	As2—O4—As1	124.65 (15)

Symmetry codes: (i) −*x*+1, *y*−1/2, −*z*+1/2; (ii) −*x*+2, −*y*+1, −*z*+1; (iii) *x*, −*y*+1/2, *z*+1/2; (iv) −*x*+2, *y*−1/2, −*z*+1/2; (v) −*x*+1, −*y*+1, −*z*+1; (vi) *x*+1, *y*, *z*.

### (NaGaAs2O7) Sodium gallium diarsenate(V) Crystal data

**Table:**

NaGa(AsO_7_)	*F*(000) = 656
*M**_r_* = 354.55	*D*_x_ = 4.418 Mg m^−^^3^
Monoclinic, *P*2_1_/*c*	Mo *K*α radiation, λ = 0.71073 Å
*a* = 6.987 (1) Å	Cell parameters from 2065 reflections
*b* = 8.266 (2) Å	θ = 3–30°
*c* = 9.677 (2) Å	µ = 17.55 mm^−^^1^
β = 107.50 (3)°	*T* = 293 K
*V* = 533.0 (2) Å^3^	Small platelets with diamond-shaped outline, colourless
*Z* = 4	0.07 × 0.07 × 0.02 mm

### (NaGaAs2O7) Sodium gallium diarsenate(V) Data collection

**Table:**

Nonius KappaCCD single-crystal four-circle diffractometer	1336 reflections with *I* > 2σ(*I*)
Radiation source: fine-focus sealed tube	*R*_int_ = 0.023
φ and ω scans	θ_max_ = 30.0°, θ_min_ = 3.1°
Absorption correction: multi-scan (HKL SCALEPACK; Otwinowski *et al.*, 2003)	*h* = −9→9
*T*_min_ = 0.373, *T*_max_ = 0.720	*k* = −11→11
3015 measured reflections	*l* = −13→13
1557 independent reflections	

### (NaGaAs2O7) Sodium gallium diarsenate(V) Refinement

**Table:**

Refinement on *F*^2^	100 parameters
Least-squares matrix: full	0 restraints
*R*[*F*^2^ > 2σ(*F*^2^)] = 0.033	*w* = 1/[σ^2^(*F*_o_^2^) + (0.060*P*)^2^ + 1.1*P*] where *P* = (*F*_o_^2^ + 2*F*_c_^2^)/3
*wR*(*F*^2^) = 0.090	(Δ/σ)_max_ < 0.001
*S* = 1.04	Δρ_max_ = 1.54 e Å^−^^3^
1557 reflections	Δρ_min_ = −1.49 e Å^−^^3^

### (NaGaAs2O7) Sodium gallium diarsenate(V) Special details

**Table:**

Geometry. All esds (except the esd in the dihedral angle between two l.s. planes) are estimated using the full covariance matrix. The cell esds are taken into account individually in the estimation of esds in distances, angles and torsion angles; correlations between esds in cell parameters are only used when they are defined by crystal symmetry. An approximate (isotropic) treatment of cell esds is used for estimating esds involving l.s. planes.

### (NaGaAs2O7) Sodium gallium diarsenate(V) Fractional atomic coordinates and isotropic or equivalent isotropic displacement parameters (Å^2^)

**Table:**

	*x*	*y*	*z*	*U*_iso_*/*U*_eq_	
Na	0.8070 (3)	0.1389 (2)	0.4691 (2)	0.0212 (4)	
Ga	0.28177 (7)	0.27455 (6)	0.49377 (5)	0.00989 (14)	
As1	0.51759 (7)	0.41477 (5)	0.29095 (5)	0.00962 (13)	
As2	0.94401 (7)	0.53707 (5)	0.29682 (5)	0.00931 (13)	
O1	0.3798 (5)	0.4129 (4)	0.1176 (3)	0.0128 (6)	
O2	0.5235 (5)	0.5967 (4)	0.3635 (4)	0.0132 (6)	
O3	0.4850 (5)	0.2586 (4)	0.3921 (4)	0.0126 (6)	
O4	0.7683 (5)	0.3827 (4)	0.2891 (4)	0.0137 (6)	
O5	0.1637 (5)	0.4423 (4)	0.3491 (3)	0.0134 (6)	
O6	0.9305 (5)	0.6834 (4)	0.4127 (4)	0.0140 (6)	
O7	0.9038 (5)	0.6014 (4)	0.1260 (3)	0.0117 (6)	

### (NaGaAs2O7) Sodium gallium diarsenate(V) Atomic displacement parameters (Å^2^)

**Table:**

	*U*^11^	*U*^22^	*U*^33^	*U*^12^	*U*^13^	*U*^23^
Na	0.0190 (10)	0.0122 (9)	0.0286 (10)	−0.0006 (8)	0.0013 (8)	0.0005 (8)
Ga	0.0130 (3)	0.0074 (2)	0.0096 (2)	0.00006 (16)	0.00367 (19)	0.00009 (16)
As1	0.0125 (2)	0.0070 (2)	0.0090 (2)	−0.00080 (15)	0.00274 (17)	0.00001 (15)
As2	0.0120 (2)	0.0073 (2)	0.0086 (2)	0.00045 (15)	0.00308 (17)	0.00032 (15)
O1	0.0175 (17)	0.0087 (14)	0.0102 (14)	−0.0003 (12)	0.0011 (12)	0.0012 (12)
O2	0.0167 (16)	0.0064 (14)	0.0164 (15)	−0.0015 (11)	0.0049 (13)	−0.0028 (12)
O3	0.0166 (16)	0.0087 (14)	0.0146 (15)	0.0022 (12)	0.0080 (13)	0.0035 (12)
O4	0.0098 (15)	0.0121 (14)	0.0198 (16)	0.0002 (12)	0.0055 (12)	0.0001 (12)
O5	0.0130 (16)	0.0124 (14)	0.0142 (15)	0.0039 (12)	0.0029 (13)	0.0043 (12)
O6	0.0188 (17)	0.0120 (15)	0.0139 (14)	−0.0035 (12)	0.0088 (13)	−0.0043 (12)
O7	0.0157 (16)	0.0116 (14)	0.0082 (13)	0.0022 (12)	0.0042 (12)	0.0033 (12)

### (NaGaAs2O7) Sodium gallium diarsenate(V) Geometric parameters (Å, º)

**Table:**

Na—O1^i^	2.289 (4)	Ga—O6^v^	1.987 (3)
Na—O6^ii^	2.360 (4)	Ga—O7^i^	2.042 (3)
Na—O3	2.364 (4)	As1—O2	1.655 (3)
Na—O7^iii^	2.468 (4)	As1—O1	1.664 (3)
Na—O7^iv^	2.479 (4)	As1—O3	1.676 (3)
Na—O4	2.624 (4)	As1—O4	1.777 (3)
Ga—O2^v^	1.940 (3)	As2—O5^vi^	1.661 (3)
Ga—O1^iii^	1.952 (3)	As2—O6	1.671 (3)
Ga—O3	1.960 (3)	As2—O7	1.678 (3)
Ga—O5	1.967 (3)	As2—O4	1.756 (3)
			
O1^i^—Na—O6^ii^	163.76 (16)	O1^iii^—Ga—O6^v^	91.80 (14)
O1^i^—Na—O3	80.93 (13)	O3—Ga—O6^v^	173.31 (13)
O6^ii^—Na—O3	114.82 (15)	O5—Ga—O6^v^	89.46 (14)
O1^i^—Na—O7^iii^	65.73 (13)	O2^v^—Ga—O7^i^	168.13 (13)
O6^ii^—Na—O7^iii^	99.98 (14)	O1^iii^—Ga—O7^i^	80.66 (14)
O3—Na—O7^iii^	126.13 (14)	O3—Ga—O7^i^	95.74 (13)
O1^i^—Na—O7^iv^	101.56 (14)	O5—Ga—O7^i^	91.80 (13)
O6^ii^—Na—O7^iv^	69.89 (12)	O6^v^—Ga—O7^i^	87.01 (13)
O3—Na—O7^iv^	137.74 (14)	O2—As1—O1	111.67 (16)
O7^iii^—Na—O7^iv^	91.40 (12)	O2—As1—O3	116.27 (16)
O1^i^—Na—O4	116.75 (14)	O1—As1—O3	116.25 (16)
O6^ii^—Na—O4	75.75 (13)	O2—As1—O4	103.93 (16)
O3—Na—O4	64.59 (11)	O1—As1—O4	105.16 (17)
O7^iii^—Na—O4	168.81 (15)	O3—As1—O4	101.46 (16)
O7^iv^—Na—O4	77.43 (12)	O5^vi^—As2—O6	111.76 (17)
O2^v^—Ga—O1^iii^	87.52 (13)	O5^vi^—As2—O7	108.37 (16)
O2^v^—Ga—O3	86.31 (14)	O6—As2—O7	113.90 (16)
O1^iii^—Ga—O3	94.67 (14)	O5^vi^—As2—O4	103.91 (16)
O2^v^—Ga—O5	100.04 (14)	O6—As2—O4	112.00 (16)
O1^iii^—Ga—O5	172.28 (14)	O7—As2—O4	106.27 (16)
O3—Ga—O5	84.37 (14)	As2—O4—As1	124.73 (19)
O2^v^—Ga—O6^v^	92.25 (14)		

Symmetry codes: (i) −*x*+1, *y*−1/2, −*z*+1/2; (ii) −*x*+2, −*y*+1, −*z*+1; (iii) *x*, −*y*+1/2, *z*+1/2; (iv) −*x*+2, *y*−1/2, −*z*+1/2; (v) −*x*+1, −*y*+1, −*z*+1; (vi) *x*+1, *y*, *z*.
